# Supplementary material for: Causal Relationship between Aging and Anorexia Nervosa: A White-Matter-Microstructure-Mediated Mendelian Randomization Analysis
Source: Biomedicines. 2024 Aug 16;12(8):1874. doi: 10.3390/biomedicines12081874 (PMC11351342; doi:10.3390/biomedicines12081874)
Supplement: Supplementary file 1 [file biomedicines-12-01874-s001.zip › Supplementary Figure/Figure S4/Singe SNP plot of MR analysis for TL to ubm-b-1691.pdf]

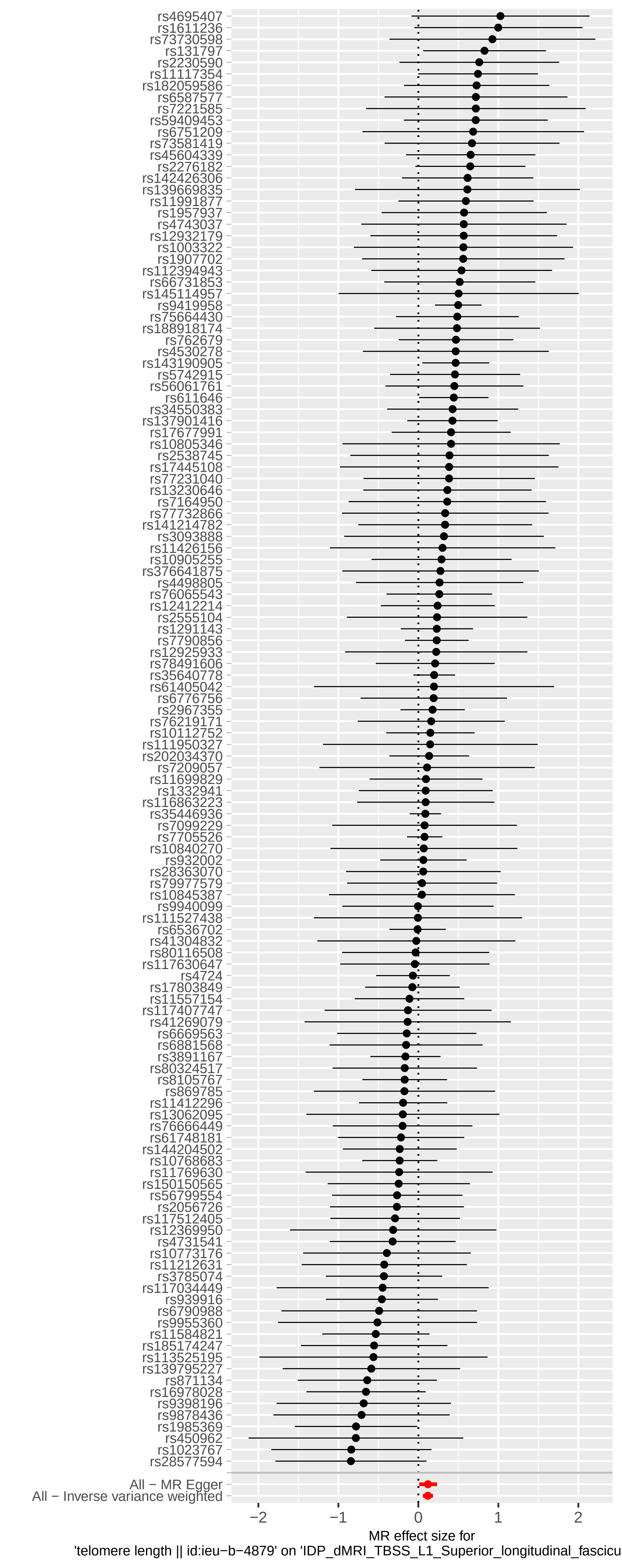

rs4695407
rs1611236
rs73730598
rs131797
rs2230590
rs11117354
rs182059586
rs6587577
rs7221585
rs59409453
rs6751209
rs73581419
rs45604339
rs2276182
rs142426306
rs139669835
rs11991877
rs1957937
rs4743037
rs12932179
rs1003322
rs1907702
rs112394943
rs66731853
rs145114957
rs9419958
rs75664430
rs188918174
rs762679
rs4530278
rs143190905
rs5742915
rs56061761
rs611646
rs34550383
rs137901416
rs17677991
rs10805346
rs2538745
rs17445108
rs77231040
rs13230646
rs7164950
rs77732866
rs141214782
rs3093888
rs11426156
rs10905255
rs376641875
rs4498805
rs76065543
rs12412214
rs2555104
rs1291143
rs7790856
rs12925933
rs78491606
rs35640778
rs61405042
rs6776756
rs2967355
rs76219171
rs10112752
rs111950327
rs202034370
rs7209057
rs11699829
rs1332941
rs116863223
rs35446936
rs7099229
rs7705526
rs10840270
rs932002
rs28363070
rs79977579
rs10845387
rs9940099
rs111527438
rs6536702
rs41304832
rs80116508
rs117630647
rs4724
rs17803849
rs11557154
rs117407747
rs41269079
rs6669563
rs6881568
rs3891167
rs80324517
rs8105767
rs869785
rs11412296
rs13062095
rs76666449
rs61748181
rs144204502
rs10768683
rs11769630
rs150150565
rs56799554
rs2056726
rs117512405
rs12369950
rs4731541
rs10773176
rs11212631
rs3785074
rs117034449
rs939916
rs6790988
rs9955360
rs11584821
rs185174247
rs113525195
rs139795227
rs871134
rs16978028
rs9398196
rs9878436
rs1985369
rs450962
rs1023767
rs28577594
All − MR Egger
All − Inverse variance weighted
−2
−1
0
1
2
MR effect size for
'telomere length || id:ieu−b−4879' on 'IDP_dMRI_TBSS_L1_Superior_longitudinal_fascicu
